# Cortical Network Alterations and Treatment‐Induced Reorganization in Panic Disorder: A Longitudinal High‐Density EEG Study

**DOI:** 10.1002/cns.71269

**Published:** 2026-11-13

**Authors:** Han Zhang, Ke Shi, Ziwei Wang, Ye Xia, Siyan Wu, Junkang Yang, Jie Feng, Kai Wang, Yuan Yang

**Affiliations:** ^1^ Department of Neurology, Tongji Hospital, Tongji Medical College Huazhong University of Science and Technology Wuhan China; ^2^ Hubei Key Laboratory of Neural Injury and Functional Reconstruction Huazhong University of Science and Technology Wuhan China; ^3^ Hubei Provincial Clinical Research Center for Stroke Wuhan China; ^4^ Hubei Technology Innovation Center for Brain‐Computer Interface Wuhan China; ^5^ Department of Neurology, Qingdao Municipal Hospital Qingdao University Qingdao China; ^6^ Department of Medical Psychology The First Affiliated Hospital of Anhui Medical University Hefei China; ^7^ Department of Neurology and Psychiatry, Tongji Hospital, Tongji Medical College Huazhong University of Science and Technology Wuhan China

**Keywords:** functional connectivity, longitudinal study, panic disorder, resting‐state electroencephalogram, topological properties

## Abstract

**Background:**

Although neuroimaging has identified network abnormalities in panic disorder (PD), the rapid and transient nature of panic symptoms highlights the need for methods with high temporal resolution. This study used high‐density electroencephalography (EEG) to investigate cortical network alterations in PD and explore their potential as translational biomarkers.

**Methods:**

This longitudinal study enrolled 52 drug‐naïve PD patients and 43 healthy controls. High‐density (128‐channel) resting‐state EEG was collected from both groups at baseline, with repeat assessments performed in PD patients after 6 weeks of venlafaxine treatment. Source‐level functional connectivity and network topology were analyzed to characterize cortical network features. Support vector machine (SVM) was used to evaluate the discriminative potential of baseline network features.

**Results:**

At baseline, PD patients showed frequency‐specific network disruptions, with enhanced connectivity within and between the default mode network, frontoparietal network, and dorsal attention network in the α band, but reduced connectivity across multiple networks in the θ and δ bands. Graph theory analysis revealed decreased global efficiency and increased characteristic path length in the α and δ bands. The SVM classifier achieved 86.32% accuracy in distinguishing PD patients from controls. Following treatment, clinical improvement was accompanied by network reorganization, including increased global efficiency and small‐worldness in the α band, and enhanced fronto‐temporal connectivity in the δ band. Exploratory brain–behavior associations did not survive FDR correction.

**Conclusions:**

PD is characterized by frequency‐specific cortical network disruptions that show dynamic reorganization following treatment. These findings highlight the potential of EEG‐derived network features as candidate markers for PD identification and treatment monitoring.

## Graphical abstract

High‐density EEG revealed frequency‐specific cortical network disruption in panic disorder, with α‐band hyperconnectivity and θ/δ‐band hypoconnectivity, followed by adaptive network reorganization after venlafaxine treatment.

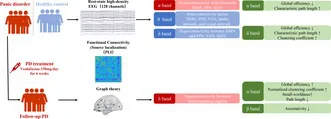

## 1 Introduction

Panic disorder (PD), a common subtype of anxiety disorders [1], is characterized by recurrent, unexpected panic attacks that reach peak intensity within minutes and significantly impair patients' social and occupational functioning [2, 3]. One prevailing hypothesis in current PD research is the neuroanatomical model, which posits that the disorder arises from aberrant information processing within brain network—a concept that has been preliminarily validated in animal models [4]. However, the exact pathophysiological mechanisms underlying PD remain unclear. Moreover, the diagnosis of PD presents significant clinical challenges due to its reliance on subjective symptom reports, somatic symptom predominance leading to frequent misdiagnosis, and high psychiatric comorbidity rates [5]. While emerging neuroimaging techniques have expanded our understanding of large‐scale brain networks, their clinical translatability remains limited, and objective biomarkers that can support diagnosis and guide treatment decisions in PD are still lacking [6, 7].

Functional magnetic resonance imaging (fMRI) studies have revealed altered connectivity patterns within and between key networks, such as the default mode network (DMN) and frontoparietal network (FPN) [8, 9, 10]. While fMRI offers high spatial resolution and enables the characterization of local neural activity and interregional functional connectivity, its limited temporal resolution restricts its ability to capture the rapid, transient dynamics of neural activity. Electroencephalography (EEG), with its excellent temporal resolution, uniquely captures these fast dynamics [11]. Unlike fMRI's indirect measurement of neural activity through blood oxygen level‐dependent signals, EEG directly measures electrical activity generated by synchronized neuronal populations, providing real‐time access to neural oscillations and their rapid fluctuations. This temporal precision is particularly crucial for understanding PD, as emerging evidence suggests that pathological processes in anxiety disorders involve rapid shifts in neural synchronization, transient network reconfigurations, and millisecond‐scale disruptions in information flow between brain regions [12]. For instance, quantitative EEG studies have reported that PD patients, with or without comorbid agoraphobia, often exhibit reduced spectral power, especially in the α‐1 band (8–11 Hz), which may reflect heightened alertness and increased non‐specific information processing demands [13]. However, traditional EEG analyses have primarily focused on localized brain activity—using metrics such as power spectral density and time–frequency analysis [13, 14]—which provide limited insight into the integrative characteristics of brain network and the interactions among distributed cortical regions.

The integration of high‐density EEG systems with source localization techniques has made it possible to examine cortical network dynamics with millisecond‐level temporal precision and improved spatial resolution. These advancements have sparked greater interest in applying graph theory and source‐level connectivity analyses to EEG data, enabling the exploration of functional network characteristics and topological properties at the cortical level [15, 16]. For example, by applying the Phase Lag Index (PLI) and graph theory to generalized social anxiety disorder patients, studies have identified increased clustering coefficients and shorter characteristic path lengths in θ‐band functional network [17]. However, in the context of PD, while some studies have preliminarily explored localized functional alterations, there remains a dearth of systematic investigations into the topological organization of the cortical functional network. Furthermore, most current PD studies adopt a cross‐sectional design [18, 19, 20], which limits insight into the longitudinal progression and potential network remodeling mechanisms underlying treatment response.

Therefore, the present study employs high‐density EEG to systematically characterize alterations in source‐level functional connectivity and topological properties of cortical functional network in PD patients. We further investigated the relationship between these network features and clinical characteristics. Additionally, through a longitudinal design, we examined changes in functional connectivity patterns and topological properties before and after a 6‐week course of pharmacological treatment and evaluated their associations with symptom improvement. This study aimed to elucidate the neurophysiological underpinnings of network reorganization in PD and provide objective biomarkers for individualized treatment strategies, thereby advancing precision psychiatry approaches.

## 2 Materials and Methods

### 2.1 Participants

This prospective cohort study was approved by the Ethics Committee of Tongji Hospital, Tongji Medical College, Huazhong University of Science and Technology (Approval Number: TJ‐IRB20221249) and was registered with the Chinese Clinical Trial Registry (Registration Number: ChiCTR2300067606, https://www.chictr.org.cn/). The study was conducted in accordance with the Declaration of Helsinki. Written informed consent was obtained from all participants voluntarily.

A total of 55 PD patients and 45 healthy controls (HCs) were initially enrolled in this study. The inclusion and exclusion criteria were as follows:

#### 2.1.1 Inclusion Criteria

PD patients: (1) Meeting the diagnostic criteria for PD according to the Diagnostic and Statistical Manual of Mental Disorders, Fifth Edition (DSM‐5) [21]; (2) Having experienced at least two full‐blown panic attacks within the past 2 weeks; (3) No history of pharmacological treatment, cognitive behavioral therapy, or other forms of psychotherapy; (4) Aged between 18 and 65 years; (5) Right‐handed; (6) Mini‐Mental State Examination (MMSE) score of ≥ 24.

HCs: (1) Aged between 18 and 65 years; (2) Right‐handed; (3) MMSE score ≥ 24; (4) Hamilton Depression Rating Scale score < 7.

#### 2.1.2 Exclusion Criteria

Participants were excluded if they met any of the following conditions: (1) History of other psychiatric disorders or substance abuse, including major depressive disorder, bipolar disorder, schizophrenia spectrum disorders, or substance use disorders; individuals with comorbid generalized anxiety disorder (GAD) were permitted if PD was the primary diagnosis and panic attacks were the predominant clinical concern; (2) Previous use of antipsychotic or psychotropic medications; (3) History of traumatic brain injury, central nervous system diseases, or other severe physical illnesses; (4) Evidence of central nervous system abnormalities on routine neuroimaging; (5) Contraindications to MRI or EEG examinations; (6) Pregnancy or lactation.

All participants underwent demographic data collection, neuropsychological assessments, and resting‐state EEG recordings at baseline. Subsequently, patients with PD received venlafaxine hydrochloride for 6 weeks. Venlafaxine was administered at 75 mg once daily during the first 2 weeks and was then increased to 150 mg once daily for the subsequent 4 weeks. Telephone follow‐up was conducted every 2 weeks during treatment to assess medication use, dose, treatment continuity, and any self‐initiated medication changes or discontinuation. At the end of week 6, patients returned for an in‐person follow‐up visit and underwent repeat neuropsychological assessments and EEG recordings. Patients who discontinued venlafaxine on their own or switched to another medication without following the study treatment protocol were considered non‐adherent, whereas patients who were lost to follow‐up were considered unable to complete the longitudinal treatment protocol.

### 2.2 Clinical and Neuropsychological Assessment

Demographic information including gender, age, and years of education were collected from all participants. Clinical characteristics were obtained from PD patients, encompassing illness duration, onset pattern, panic attack frequency in the preceding 2 weeks, and relevant medical, personal, and family histories. All participants completed a series of neuropsychological assessments to quantify their clinical symptoms and psychological status. The Panic Disorder Severity Scale (PDSS) [22] was used to evaluate the severity and impact of PD symptoms, while the Hamilton Anxiety Rating Scale (HAMA) [23] was employed to assess anxiety levels.

### 2.3 EEG Data Acquisition

Resting‐state EEG data were recorded using a BrainAmp MR EEG system (Brain Products, Munich, Germany) equipped with a 128‐channel Ag/AgCl electrode cap following the extended international 10–20 system. Continuous EEG signals were recorded via the Brain Vision Recorder software. The parameters of resting‐state EEG data were provided in the Supplementary Materials.

### 2.4 EEG Data Processing

#### 2.4.1 EEG Data Preprocessing

EEG preprocessing was performed using the EEGLAB toolbox (https://sccn.ucsd.edu/eeglab/index.php/) in MATLAB 2022b [24]. The main processing steps including re‐referencing, filtering, downsampling, segmentation, bad channel/segment handling, independent component analysis, and quality control were detailed in the Supplementary Materials.

#### 2.4.2 Source Localization

Source localization was conducted using the Minimum Norm Estimation method, and cortical signals were parcellated according to the Desikan–Killiany atlas with the Brainstorm toolbox (https://neuroimage.usc.edu/brainstorm/) in MATLAB 2022b [25], in order to minimize spurious connectivity arising from volume conduction effects. Detailed source‐localization procedures are described in the Supplementary Materials, and the full list of the 68 cortical regions used for source‐space parcellation is provided in Supporting Information Table 1.

#### 2.4.3 Functional Connectivity

Functional connectivity was computed using the PLI method, following procedures described in previous studies [26, 27]. The PLI ranges from 0 to 1, where a value of 0 indicates complete asynchrony between the EEG signals of two brain regions, and a value of 1 indicates perfect synchrony in their phase relationships. Functional connectivity matrices were computed for each subject and segmented into specific frequency bands. The resulting matrices corresponding to the α (8–13 Hz), β (13–30 Hz), θ (4–8 Hz), and δ (0.5–4 Hz) bands were extracted and used for subsequent statistical and graph‐theoretical analyses.

#### 2.4.4 Graph Theory Analysis

Graph theory analysis was performed using the GRETNA toolbox implemented in MATLAB 2022b. Topological properties of the cortical functional network were computed separately for each frequency band. The threshold of sparsity was set to range from 0.06 to 0.50 (with a step size of 0.01). Both global and nodal metrics of the cortical functional network were analyzed. Global metrics included small‐worldness (*σ*), normalized clustering coefficient, normalized characteristic path length, clustering coefficient, characteristic path length, global efficiency, local efficiency, assortativity, hierarchy, and synchronizability. Nodal metrics included degree centrality, betweenness centrality, nodal efficiency, nodal local efficiency, nodal shortest path length, and nodal clustering coefficient. The detailed information regarding the global and nodal properties in the graph theoretical analysis can be found in Appendix 1.

### 2.5 Support Vector Machine (SVM)

To evaluate the diagnostic potential of EEG‐derived network features for PD identification, we implemented a machine learning pipeline using SVM classification. EEG data from PD patients and HCs were first preprocessed to construct a functional connectivity matrix and compute corresponding graph metrics. Features showing significant group differences were identified through statistical analysis, followed by recursive feature elimination to select the most informative subset. The SVM classifier was evaluated using 10‐fold cross‐validation, with classification metrics including accuracy, sensitivity, specificity, and the area under the curve (AUC).

### 2.6 Statistical Analysis

Baseline Stage: Statistical analyses of demographic and clinical characteristics were performed using SPSS version 24.0. Categorical variables were presented as percentages, while continuous variables were presented as mean ± standard deviation or interquartile range, as appropriate. Group comparisons between the PD and HC groups were conducted using the chi‐square test for categorical variables, and either two‐sample *t*‐test (parametric) or Mann–Whitney *U* test (non‐parametric) for continuous variables, depending on the distribution. The Kolmogorov–Smirnov test was used to assess the normality of data distribution. Statistical significance was set at *p* < 0.05.

For functional connectivity analysis, a two‐sample *t*‐test was first used to compare connectivity strength between groups. Multiple comparison correction was performed using the Network‐Based Statistic (NBS) method. A primary statistical threshold of *p* < 0.001 was used to identify potential abnormal connections in the connectivity matrix. NBS was then applied using a non‐parametric permutation test to determine the significance of subnetworks, with a corrected threshold of *p* < 0.05.

For graph theory analysis, group differences in global metrics were assessed using a two‐sample *t*‐test, with a significance level of *p* < 0.05. Nodal metrics were also compared using a two‐sample *t*‐test, followed by false discovery rate (FDR) correction for multiple comparisons, with an adjusted significance level of *p* < 0.05.

In correlation analysis, partial correlation analysis was conducted to examine the relationships between these altered metrics and neuropsychological characteristics in the PD group. The significance level was set at *p* < 0.05.

Longitudinal Follow‐Up Stage: Paired *t*‐tests (parametric) or Wilcoxon signed‐rank tests (non‐parametric) were used to evaluate changes in scale scores before and after treatment. A significance level of *p* < 0.05 was applied.

For functional connectivity, paired *t*‐tests were used to compare pre‐ and post‐treatment connectivity strengths, and NBS was applied for multiple comparison correction. The primary and corrected significance thresholds were set as described previously.

For graph theory analysis, paired *t*‐tests were applied to compare global network metrics between pre‐ and post‐treatment conditions. For nodal metrics, paired *t*‐tests were also conducted, and multiple comparisons were corrected using the FDR method. The significance threshold for *p*‐values remained consistent with previous analyses.

Correlation analyses were performed using Pearson correlation to assess the relationship between changes in abnormal functional connectivity and topological properties and changes in clinical scale scores, with the significance level set at *p* < 0.05.

## 3 Results

### 3.1 Baseline Stage

#### 3.1.1 Demographic Data and Clinical Characteristics

Due to poor EEG data quality, 3 participants from the PD group and 2 from the HC group were excluded. As a result, 52 PD patients and 43 HCs were ultimately included in the final analysis.

Supplementary Table 1 presents detailed demographic and clinical characteristics of the PD and HC groups. The results showed no significant differences between the two groups in terms of age, sex, or years of education. However, the PD group had significantly higher HAMA scores compared to the HC group (*p* < 0.001).

#### 3.1.2 Functional Connectivity

At baseline, patients with PD demonstrated significant alterations in functional connectivity across multiple frequency bands, primarily involving frontal, parietal, temporal, and limbic regions (Figure 1 and Supplementary Table 2). In the α band, enhanced connectivity was primarily observed among the left superior parietal cortex, left temporal pole, right caudal anterior‐cingulate cortex, left superior frontal gyrus, and right rostral middle frontal gyrus. This increase reflected stronger connectivity within the DMN and FPN, as well as between these networks and the dorsal attention network (DAN). In the θ band, decreased connectivity was found among regions including the left isthmus‐cingulate cortex, left rostral middle frontal gyrus, left rostral anterior cingulate cortex, left insula cortex, left superior frontal gyrus, left pericalcarine cortex, right isthmus‐cingulate gyrus, left medial orbitofrontal cortex, right lateral orbitofrontal cortex, right parahippocampal gyrus, and right frontal pole, involving the DMN, FPN, ventral attention network (VAN), limbic network, and visual network. In the δ band, reduced connectivity was primarily characterized by significantly reduced connectivity between the DMN and other networks, including the FPN, the VAN, and the somatomotor network (SMN). This reduction involved key regions such as the left lateral orbitofrontal cortex, left medial orbitofrontal cortex, left rostral anterior cingulate cortex, right caudal middle frontal gyrus, right inferior temporal gyrus, and right paracentral lobule. In the β band, no significant differences in functional connectivity were observed after multiple comparison correction (all corrected *p* > 0.05).

**FIGURE 1:**
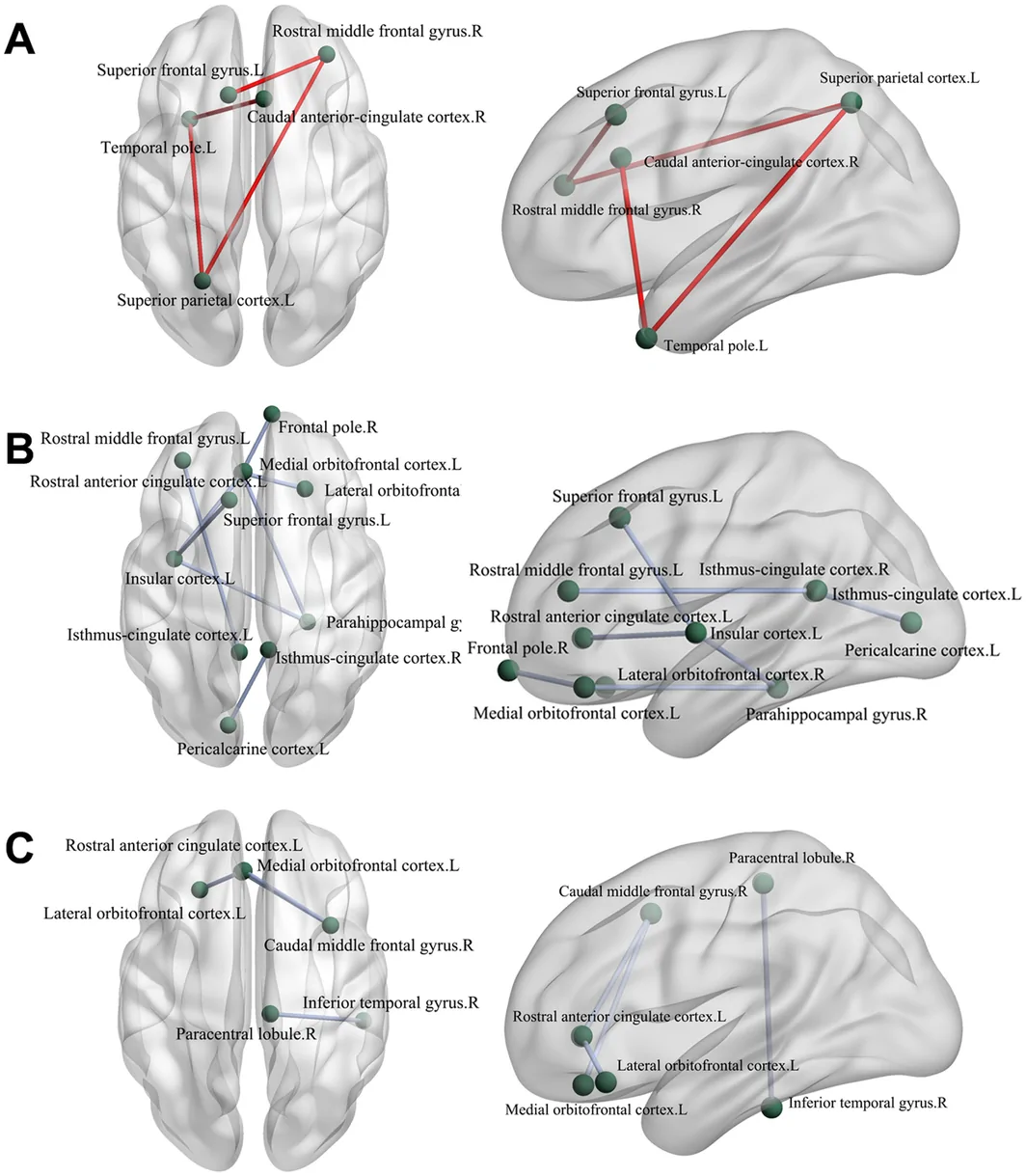
Group differences in cortical functional connectivity between PD and HC across different frequency bands. (A) Abnormal functional connectivity in the *α* band. (B) Abnormal functional connectivity in the θ band. (C) Abnormal functional connectivity in the δ band. Red lines indicate increased functional connectivity, while blue lines indicate decreased functional connectivity.

#### 3.1.3 Graph Theory Analysis

To further elucidate the topological abnormalities underlying altered brain connectivity in PD, we examined both global and nodal properties of the cortical functional network across frequency bands using graph theory analysis. In terms of global topological properties, the PD group exhibited decreased global efficiency and increased characteristic path length in the α band. In the δ band, decreased global efficiency, along with increased clustering coefficient and characteristic path length, were observed in the PD group (Table 1 and Supplementary Table 3).

**TABLE 1:** Group differences in topological properties of cortical functional network between PD and HC.

Graph measures	Mean ± SD		*T* value	*p*
	HC group	PD group		
*α* band				
Global efficiency	0.243 ± 0.019	0.235 ± 0.020	−2.120	0.037*
Characteristic path length	0.880 ± 0.105	0.926 ± 0.106	2.088	0.040*
*θ* band				
Nodal efficiency				
*Fusiform gyrus.L*	0.230 ± 0.064	0.267 ± 0.035	3.399	0.001*
*δ* band				
Global efficiency	0.264 ± 0.005	0.260 ± 0.008	−3.403	0.001*
Clustering coefficient	0.174 ± 0.014	0.181 ± 0.017	2.045	0.044*
Characteristic path length	0.767 ± 0.022	0.787 ± 0.033	3.453	0.001*
Nodal betweenness centrality				
*Pars opercularis of inferior Frontal gyrus.L*	17.792 ± 20.476	8.160 ± 8.518	−2.885	0.006*
*Superior Frontal gyrus.L*	15.573 ± 14.370	8.312 ± 8.509	−2.917	0.005*
*Banks superior temporal sulcus.R*	17.129 ± 14.186	8.718 ± 5.956	−3.630	0.001*
*Inferior temporal gyrus.R*	17.125 ± 14.186	8.374 ± 5.956	−2.963	0.004*

*Note:* Positive *T*‐values indicate enhanced network topological properties, while negative *T*‐values indicate reduced network topological properties.

* The results were statistically significant (*p* < 0.05).

Regarding nodal topological properties, the PD group exhibited increased nodal efficiency in the left fusiform gyrus within the θ band. Additionally, decreased betweenness centrality was observed in several cortical regions within the δ band, including the left pars opercularis of inferior frontal gyrus, left superior frontal gyrus, right banks superior temporal sulcus, and right inferior temporal gyrus (Table 1).

#### 3.1.4 Correlation Analysis

To explore the clinical significance of the observed network alterations, we assessed correlations between EEG‐derived network metrics and clinical symptom scores in the PD group. We found that the HAMA scores were positively correlated with the functional connectivity between left medial orbitofrontal cortex and right frontal pole in the θ band (Figure 2A). The PDSS scores were positively correlated with the functional connectivity between left medial orbitofrontal cortex and right lateral orbitofrontal cortex in the θ band (Figure 2B), and negatively correlated with the functional connectivity between right inferior temporal gyrus and right paracentral lobule in the δ band (Figure 2C). Additionally, HAMA scores were positively correlated with the clustering coefficient of the cortical functional network in the δ band (Figure 2D) and with the betweenness centrality of the left pars opercularis of the inferior frontal gyrus (Figure 2E). Although several correlations between altered network metrics and clinical scores were observed, none survived FDR correction.

**FIGURE 2:**
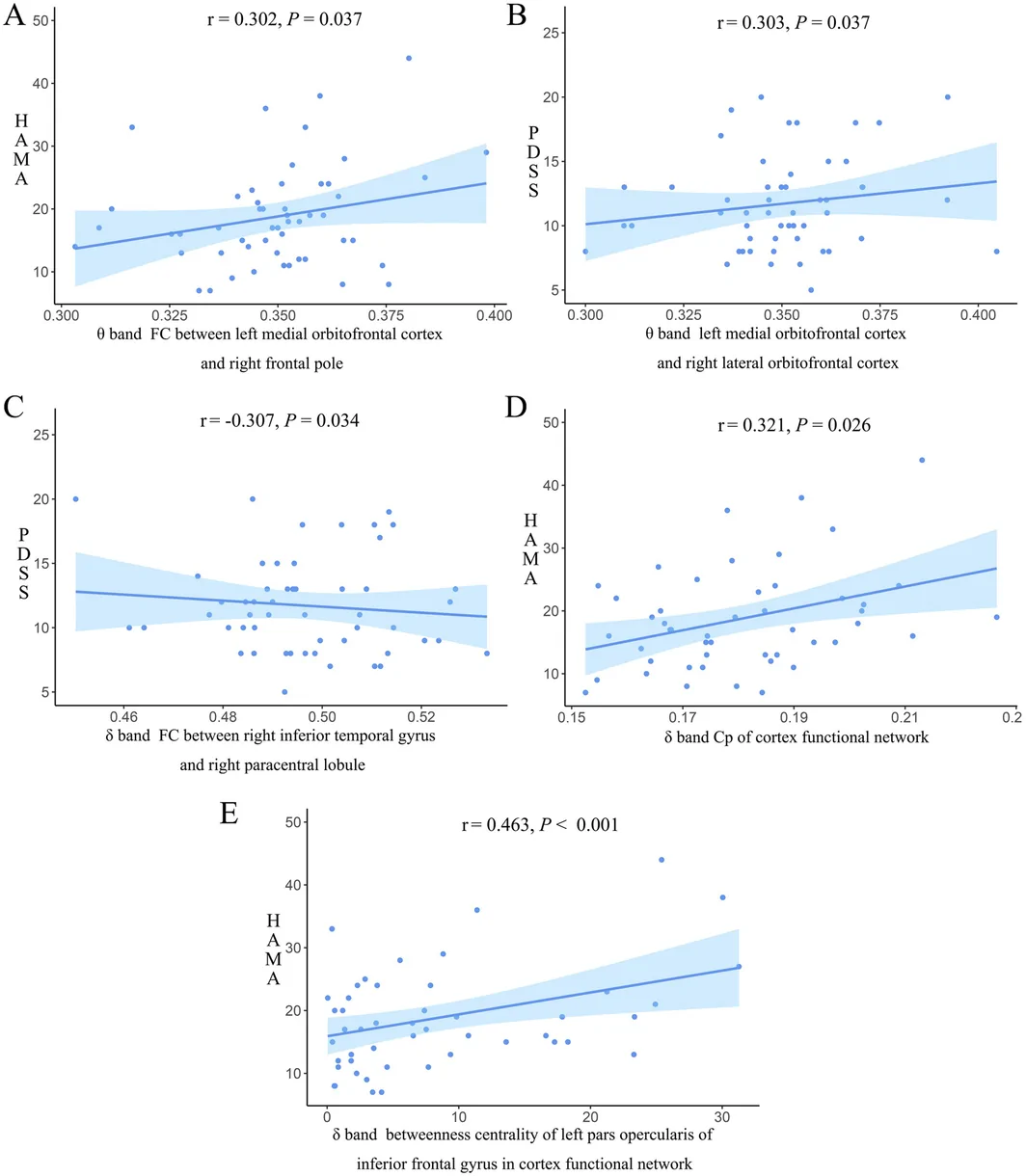
Correlations between abnormal functional connectivity/topological properties and neuropsychological test scores in patients with PD.

#### 3.1.5 SVM

To evaluate the discriminative potential of altered EEG‐based network features in identifying PD patients, we conducted a SVM classification analysis using functional connectivity and graph theory metrics. A total of 26 features that showed significant between‐group differences at baseline were initially included. Through feature selection, the top 20 features were selected to construct the classification model. The model showed strong performance during cross‐validation, achieving an accuracy of 0.8632, sensitivity of 0.907, specificity of 0.8269, and an AUC of 0.8810. More details were present in Appendix 1.

### 3.2 Longitudinal Follow‐Up Stage

#### 3.2.1 Clinical Characteristics

To assess the effects of pharmacological intervention over time, follow‐up EEG and clinical assessments were conducted after 6 weeks of treatment. During the follow‐up period, 9 patients were excluded due to refusal to take medication, and another 11 patients were excluded due to irregular medication adherence or self‐discontinuation of treatment. Ultimately, 32 patients underwent follow‐up EEG assessments after receiving pharmacological treatment, among whom 1 patient was excluded due to poor EEG data quality at the follow‐up stage. Consequently, a total of 31 patients with PD completed the 6‐week follow‐up and were included in the subsequent data analysis. Comparisons of baseline demographic and clinical characteristics between follow‐up completers and non‐completers showed no significant differences in sex, age, years of education, illness duration, or HAMA scores (all *p* > 0.05), whereas baseline PDSS scores were significantly higher in follow‐up completers than in non‐completers [12 (10, 15) vs. 10 (8, 12), *p* = 0.016]. Among patients included in the longitudinal analysis, both HAMA and PDSS scores were significantly reduced after treatment (*p* < 0.001), indicating a marked improvement in anxiety levels and disease severity of PD following pharmacological intervention (Supplementary Table 4).

#### 3.2.2 Functional Connectivity

To examine treatment‐induced changes in brain network integration, functional connectivity was compared between pre‐ and post‐treatment EEG recordings. After multiple comparison correction, patients with PD exhibited significantly enhanced functional connectivity in multiple fronto‐temporal regions within the δ band. As shown in Figure 3 and Supplementary Table 5, the increased abnormal connectivity primarily involved the left medial orbitofrontal cortex, left insula cortex, right lateral orbitofrontal cortex, right postcentral gyrus, right precentral gyrus, bilateral superior temporal gyri, right transverse temporal gyrus, and right pars triangularis of inferior frontal gyrus.

**FIGURE 3:**
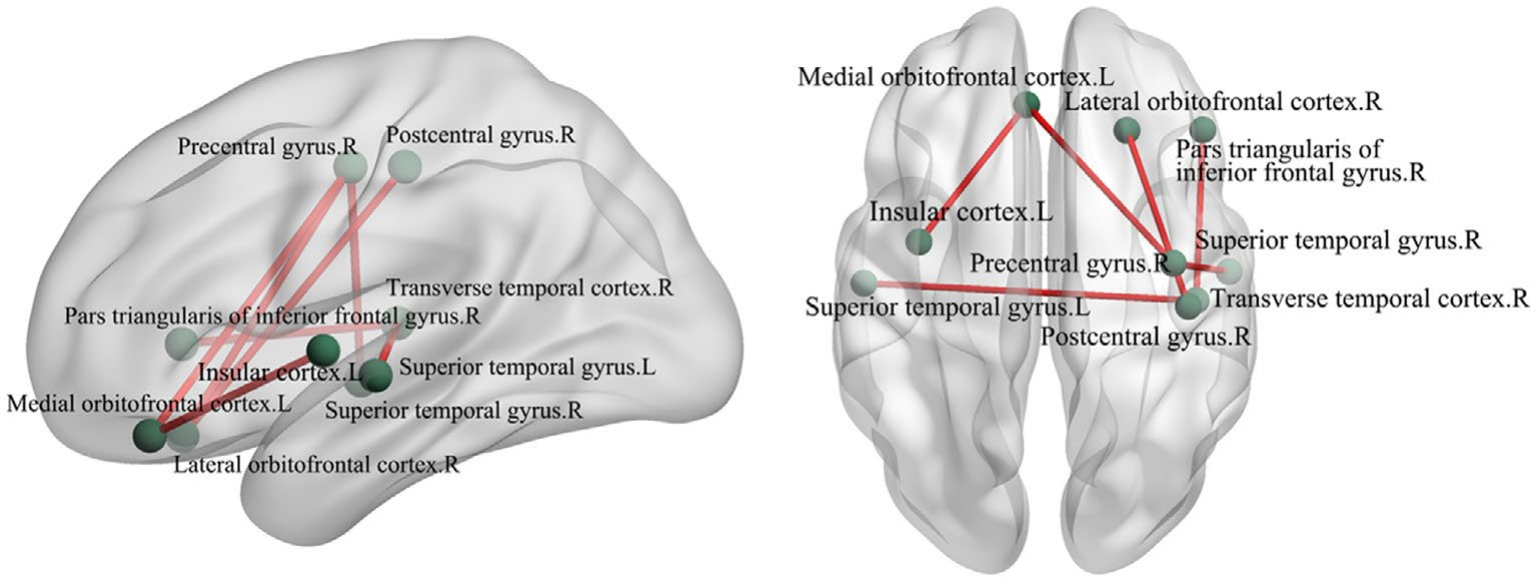
Group differences in cortical functional connectivity between pre‐ and post‐treatment in PD patients in the δ band. Red lines indicate increased functional connectivity.

#### 3.2.3 Graph Theory Analysis

Graph theory analysis revealed significant post‐treatment reorganization in cortical network topology. As shown in Table 2 and Supplementary Table 6, alterations in global topological properties of cortical functional network were observed in the *α* and *β* bands. In the α band, patients with PD exhibited increased global efficiency, normalized clustering coefficient, and small‐worldness, along with a significant reduction in characteristic path length. In the β band, the assortativity of cortical functional network was decreased in the PD group.

**TABLE 2:** Differences in Topological Properties of the Cortical Functional Network Between Pre‐ and Post‐Treatment in PD Patients.

Graph measures	Mean ± SD		*T* value	*p*
	Pre‐treatment group	Post‐treatment group		
*α* band				
Global efficiency	0.233 ± 0.019	0.244 ± 0.019	3.562	0.001*
Normalized clustering coefficient	0.371 ± 0.034	0.386 ± 0.039	2.802	0.009*
Characteristic path length	0.935 ± 0.098	0.885 ± 0.110	−2.759	0.010*
Small‐worldness	0.367 ± 0.032	0.382 ± 0.039	2.739	0.010*
Nodal degree centrality				
*Pars orbitalis of inferior frontal gyrus.L*	11.517 ± 2.556	9.625 ± 3.256	−2.998	0.005*
*Superior frontal gyrus.L*	13.334 ± 2.785	9.895 ± 3.968	−4.179	< 0.001*
*Superior parietal cortex.L*	13.225 ± 3.471	10.827 ± 4.065	−2.915	0.007*
*Temporal pole.L*	11.194 ± 4.100	8.683 ± 3.704	−3.061	0.005*
*Caudal anterior‐cingulate cortex.R*	12.648 ± 1.718	10.557 ± 2.535	−4.319	< 0.001*
*Caudal middle frontal gyrus.R*	4.210 ± 4.675	8.203 ± 6.729	3.030	0.005*
*Precentral gyrus.R*	1.764 ± 2.204	4.894 ± 2.874	5.155	< 0.001*
*Precuneus cortex.R*	2.128 ± 2.238	4.824 ± 3.779	3.681	< 0.001*
*Temporal pole.R*	10.500 ± 2.563	8.264 ± 3.179	−2.987	0.006*
Nodal clustering coefficient				
*Pericalcarine cortex.R*	0.075 ± 0.062	0.131 ± 0.070	3.852	< 0.001*
*Precentral gyrus.R*	0.082 ± 0.074	0.147 ± 0.063	4.389	0.002*
Nodal efficiency				
*Precuneus cortex.L*	0.179 ± 0.067	0.218 ± 0.068	3.405	0.002*
*Superior frontal gyrus.L*	0.292 ± 0.021	0.265 ± 0.045	−3.066	0.005*
*Transverse temporal cortex.L*	0.222 ± 0.060	0.245 ± 0.045	2.814	0.009*
*Caudal anterior‐cingulate cortex.R*	0.288 ± 0.139	0.276 ± 0.019	−3.040	0.005*
*Caudal middle frontal gyrus.R*	0.148 ± 0.105	0.234 ± 0.082	3.811	< 0.001*
*Pericalcarine cortex.R*	0.112 ± 0.075	0.168 ± 0.087	2.878	0.007*
*Precentral gyrus.R*	0.115 ± 0.080	0.206 ± 0.061	6.652	< 0.001*
*Precuneus cortex.R*	0.127 ± 0.078	0.198 ± 0.066	4.780	< 0.001*
*Superior temporal gyrus.R*	0.249 ± 0.053	0.269 ± 0.047	2.955	0.006*
Nodal local efficiency				
*Pericalcarine cortex.R*	0.101 ± 0.084	0.177 ± 0.094	3.703	< 0.001*
*Precentral gyrus.R*	0.104 ± 0.093	0.209 ± 0.085	5.483	< 0.001*
*Precuneus cortex.R*	0.127 ± 0.092	0.196 ± 0.090	3.389	0.002*
*β* band				
Assortativity	0.477 ± 1.109	−0.125 ± 0.941	−2.428	0.021*
Nodal betweenness centrality				
*Precentral gyrus.R*	4.501 ± 4.559	11.270 ± 9.882	4.057	< 0.001*
Nodal degree centrality				
*Superior parietal cortex.L*	11.479 ± 2.680	8.960 ± 4.149	−3.213	0.003*
*Caudal anterior‐cingulate cortex.R*	11.010 ± 2.931	8.927 ± 2.632	−3.330	0.002*
*Caudal middle frontal gyrus.R*	3.481 ± 2.687	8.228 ± 5.129	4.796	< 0.001*
*Lateral occipital cortex.R*	11.173 ± 2.859	8.533 ± 4.053	−3.471	0.002*
*Precentral gyrus.R*	5.730 ± 2.639	8.458 ± 3.269	3.657	< 0.001*
Nodal efficiency				
*Caudal anterior‐cingulate cortex.R*	0.288 ± 0.021	0.271 ± 0.023	−3.399	0.002*
*Caudal middle frontal gyrus.R*	0.191 ± 0.051	0.255 ± 0.054	5.397	< 0.001*
*Lateral occipital cortex.R*	0.289 ± 0.024	0.263 ± 0.042	−3.390	0.002*
Nodal local efficiency				
*Caudal middle frontal gyrus.R*	0.170 ± 0.095	0.256 ± 0.048	4.858	< 0.001*
*δ* band				
Nodal clustering coefficient				
*Insular cortex.R*	0.205 ± 0.052	0.173 ± 0.052	−4.194	< 0.001*

*Note:* Positive *T*‐values represent enhanced network topological properties after treatment, while negative *T*‐values represent reduced network topological properties after treatment.

* The results were statistically significant (*p* < 0.05, FDR corrected).

In terms of nodal topological properties, significant alterations were observed in the cortical functional network of PD patients following pharmacological treatment. Changes in nodal metrics—including degree centrality, nodal clustering coefficient, nodal efficiency, nodal local efficiency, and betweenness centrality—were identified across multiple brain regions within the DMN, FPN, and SMN in the α, β, and δ bands. Detailed results are presented in Table 2.

#### 3.2.4 Correlation Analysis

The clinical significance of network reorganization was further explored by examining Pearson correlations between changes in symptom severity (HAMA and PDSS scores) and nodal topological alterations across key cortical regions (Figure 4). Several nominal associations were observed across the *α* and *β* bands. Specifically, changes in HAMA scores were negatively correlated with alterations in degree centrality of the right precuneus cortex, nodal efficiency of the left transverse temporal cortex, and nodal efficiency of the right precuneus cortex in the α band (Figure 4A,C,D), but positively correlated with changes in the degree centrality of the right temporal pole (Figure 4B). In the β band, changes in HAMA scores were negatively correlated with changes in the nodal local efficiency of the right caudal middle frontal gyrus (Figure 4E).

**FIGURE 4:**
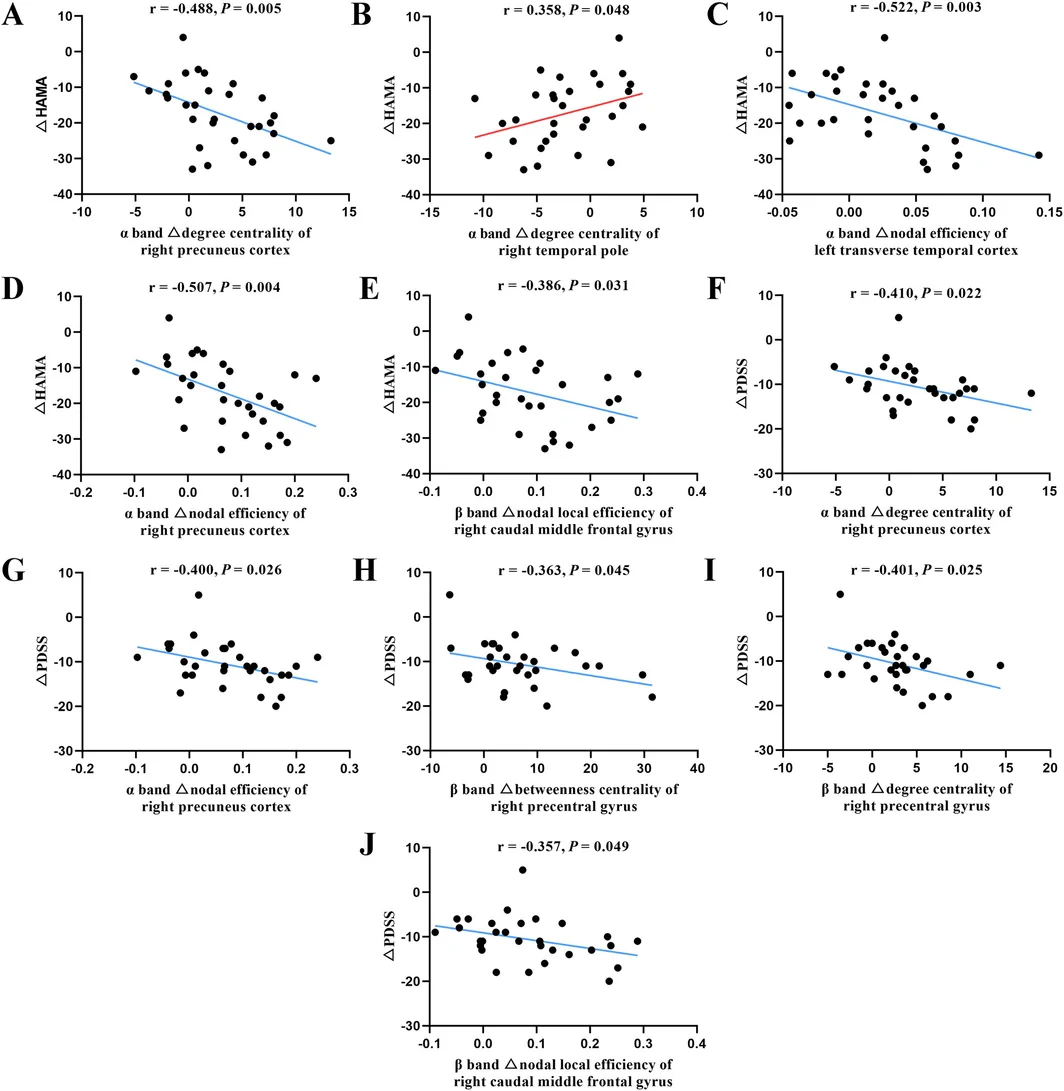
Correlations between changes in abnormal topological properties of functional network and changes in neuropsychological test scores after treatment in patients with PD.

Regarding PDSS scores, changes were negatively correlated with both degree centrality and nodal efficiency of the right precuneus cortex in the α band (Figure 4F,G). In the β band, PDSS score changes were negatively correlated with changes in betweenness centrality and degree centrality of the right precentral gyrus, as well as nodal local efficiency of the right caudal middle frontal gyrus (Figure 4H–J). However, none of these associations survived FDR correction, and they should therefore be considered exploratory.

## 4 Discussion

This longitudinal study employed high‐density EEG to characterize cortical functional network dynamics in panic disorder, revealing frequency‐specific alterations at baseline and treatment‐induced reorganization associated with clinical improvement. Our findings provide novel insights into the temporal dynamics of PD pathophysiology and offer potential biomarkers for diagnosis and treatment monitoring.

At baseline, PD patients exhibited a distinctive pattern of multi‐frequency network dysfunction characterized by opposing alterations across different frequency bands. This frequency‐dependent pattern reveals a fundamental disruption in the temporal hierarchy of neural oscillations, which may underlie the core symptoms of PD. In the α band, which reflects cortical idling and relaxed wakefulness [28], we observed enhanced functional connectivity within the DMN, FPN, and DAN, which plays a critical role in the orienting and sustaining of attention, particularly in processing external visuospatial information and supporting dynamic attentional shifts [29]. The abnormal enhancement of connectivity among these networks, accompanied by decreased global efficiency and increased path length [30] suggests inefficient information processing despite compensatory hyperconnectivity, potentially reflecting the hypervigilance characteristic of PD. The DMN‐FPN dysconnectivity may underlie the impaired balance between internal self‐referential processing and external task‐oriented attention [31, 32].

In contrast, the θ and δ band, PD patients exhibited predominant reduced connectivity across multiple networks, primarily involving the DMN, FPN, and VAN. The observed widespread reductions in functional connectivity among these networks suggest a marked impairment in the brain's integrative processing capacity in individuals with PD. At the network level, increased clustering coefficients alongside decreased betweenness centrality point to a shift toward more locally clustered but less globally efficient communication [33]. These patterns may reflect a compensatory mechanism aimed at preserving regional processing but could also contribute to elevated anxiety, as supported by positive correlations between anxiety levels and both global clustering and the centrality of the left posterior inferior frontal gyrus.

Collectively, these results indicate a frequency‐dependent network dysfunction in PD: with α‐band hyperconnectivity suggesting overactive internal monitoring, and θ/δ hypoconnectivity reflecting deficits in emotional and interoceptive integration. This imbalance may underlie the impaired ability of PD patients to flexibly switch between internal states and external demands, a critical mechanism for adaptive emotional regulation.

Following 6 weeks of venlafaxine treatment, we observed significant reorganization of cortical functional network alongside marked clinical improvement. The α band showed the most pronounced changes, with increased global efficiency and small‐worldness, suggesting restoration of optimal network topology. Key nodes, such as the precuneus and temporal regions, demonstrated treatment‐related alterations in centrality, some of which showed nominal associations with changes in symptom severity. The right temporal pole, in particular, showed reduced hub status following treatment—a change that may reflect a decrease in maladaptive emotional processing and rumination [34, 35]. In the δ band, we observed strengthened connectivity across DMN, SMN, and VAN, suggesting compensatory reorganization rather than a simple reversal of baseline deficits. The right insula, a region central to interoceptive awareness, exhibited decreased local clustering post‐treatment, potentially reflecting reduced hypervigilance to bodily sensations [36, 37]. This finding aligns with recent evidence that successful anxiety treatment may involve “de‐hubbing” of hyperactive emotional processing nodes [38, 39, 40]. Collectively, these changes demonstrate frequency‐specific treatment‐related network reorganization: enhanced δ band connectivity may reflect improved emotional regulation and interoceptive processing, whereas concurrent α‐band topological changes may indicate improved efficiency of attentional and arousal‐related networks. These findings suggest that treatment‐related network changes in PD may extend beyond a simple reversal of baseline abnormalities and potentially involve broader adaptive reorganization. The dynamic reorganization observed reinforces the concept of neural plasticity and highlights EEG‐derived metrics as sensitive markers of treatment effects. Importantly, although exploratory correlation analyses revealed several nominal associations between network changes and symptom improvement, none survived FDR correction. These brain–behavior associations should therefore be interpreted cautiously and require replication in larger independent samples.

These EEG findings show a certain degree of consistency with recent fMRI studies of PD. One study reported altered degree centrality and functional connectivity involving the anterior cingulate, temporal, and occipital regions, together with treatment‐related functional changes following pharmacotherapy [41]. Another study demonstrated disrupted network homogeneity across the FPN, DMN, SMN, limbic, and VAN, as well as network reorganization after treatment [42]. Together, these fMRI findings provide partially convergent evidence with our observation of distributed network abnormalities and treatment‐related reorganization across multiple cortical networks. Importantly, our EEG results further highlight the frequency‐dependent nature of these alterations, suggesting that EEG and fMRI may provide complementary temporal and spatial perspectives on network dysfunction and plasticity in PD.

Our findings provide early‐stage evidence supporting the use of EEG‐derived network features as practical biomarkers in panic disorder. The observed frequency‐specific alterations in functional network topology not only reflect underlying neurophysiological dysfunctions but also align with the established roles of different oscillatory bands: α band hyperconnectivity corresponds to heightened vigilance and attentional bias, while reduced θ/δ band connectivity points to deficits in emotional integration and interoceptive regulation. These patterns demonstrate that EEG uniquely captures oscillatory dynamics that cannot be adequately resolved by other neuroimaging modalities such as fMRI, thereby underscoring its distinctive value. Importantly, the significant reorganization of these networks following pharmacological intervention highlights neural plasticity and suggests that EEG can serve as a dynamic, real‐time indicator of therapeutic efficacy. Moreover, the high classification accuracy of SVM emphasizes the potential of EEG‐derived network markers for the early identification of PD. Looking forward, integrating these frequency‐specific biomarkers into real‐time EEG feedback systems or digital therapeutics could further strengthen their translational potential and enable scalable, individualized treatment monitoring in clinical practice.

This study has several key strengths. It is the first longitudinal analysis of cortical functional network in PD using high‐density EEG, allowing precise tracking of fast neural dynamics relevant to panic symptoms. The integration of source‐level connectivity and graph theory enabled a detailed, multiscale characterization of network alterations. Importantly, the within‐subject longitudinal design reduced individual variability and strengthened the link between neural changes and symptom improvement.

Despite the valuable findings, several limitations should be noted. First, the relatively small and homogeneous sample limits the generalizability of the results. In addition, a proportion of patients did not complete the six‐week follow‐up, which may have introduced potential attrition bias. Although follow‐up completers and non‐completers did not differ significantly in most baseline demographic and clinical characteristics, baseline PDSS scores were significantly higher among completers. Therefore, the longitudinal findings may be more representative of patients with greater panic severity and good treatment adherence, and their generalizability to the broader PD population should be interpreted with caution. Second, this study focused solely on changes in brain network characteristics after 6 weeks of treatment. However, treatment for PD is often a long‐term process. Therefore, longitudinal follow‐up studies are warranted to track the dynamic evolution of brain networks throughout extended treatment courses. Third, the present study was based on resting‐state EEG data alone. Future research may benefit from integrating multimodal neuroimaging data, including both resting‐state and task‐based paradigms, to capture a more comprehensive temporal and spatial profile of brain network characteristics in PD. Fourth, EEG source reconstruction is inherently subject to spatial uncertainty, particularly for sources with less favorable geometry or those located near the boundaries of the source space. Although ROI‐level parcellation may reduce the influence of localization errors at individual vertices, such uncertainty cannot be completely eliminated. Finally, the observed associations between EEG‐derived network measures and clinical symptoms did not survive FDR correction. These brain–behavior associations should therefore be regarded as exploratory and interpreted with caution until replicated in larger independent samples.

## 5 Conclusion

This longitudinal EEG study reveals frequency‐specific cortical network alterations in PD: α band hyperconnectivity and θ/δ band hypoconnectivity within key regions of the DMN and FPN at baseline, with differential reorganization following treatment. The observed adaptive network changes, rather than simple restoration, correlated with clinical improvement. These findings not only enhance our understanding of the neurobiological mechanisms underlying PD but also support the use of EEG‐based network metrics as objective tools for diagnosis and treatment monitoring in clinical practice.

## Author Contributions

H.Z. and K.S. contributed equally to this work. H.Z. and K.S. designed the study and drafted the manuscript. Z.W., Y.X., and S.W. were responsible for data acquisition and preprocessing. J.Y. and J.F. performed statistical analyses and contributed to the interpretation of results. K.W. provided methodological guidance and critical revision of the manuscript. Y.Y. supervised the project, contributed to study design, and revised the manuscript for important intellectual content. All authors read and approved the final manuscript.

## Funding

The study was supported by the National Natural Science Foundation of China (82090034).

## Ethics Statement

This prospective cohort study was approved by the Ethics Committee of Tongji Hospital, Tongji Medical College, Huazhong University of Science and Technology (Approval Number: TJ‐IRB20221249) and was registered with the Chinese Clinical Trial Registry (Registration Number: ChiCTR2300067606, https://www.chictr.org.cn/).

## Consent

The authors have nothing to report.

## Conflicts of Interest

The authors declare no conflicts of interest.

## Supporting information


**Supporting Information Table 1:** Cortical regions defined by the Desikan–Killiany atlas used for source‐space parcellation.

**Supplementary Table 1:** The demographic characteristics, clinical information of all participants.
**Supplementary Table 2:** Group Differences in Cortical Functional Connectivity between PD and HC.
**Supplementary Table 3:** Group Differences in Global Topological Properties of Cortical Functional Network between PD and HC.
**Supplementary Table 4:** Pre‐ and Post‐treatment Clinical Data in PD Patients.
**Supplementary Table 5:** Group Differences in Cortical Functional Connectivity Between Pre‐ and Post‐Treatment in PD Patients.
**Supplementary Table 6:** Differences in Global Topological Properties of the Cortical Functional Network Between Pre‐ and Post‐Treatment in PD Patients.

## Data Availability

The data that support the findings of this study are available on request from the corresponding author. The data are not publicly available due to privacy or ethical restrictions.
