# Supplementary material for: Cortical Network Alterations and Treatment‐Induced Reorganization in Panic Disorder: A Longitudinal High‐Density EEG Study
Source: CNS Neurosci Ther. 2026 Nov 13;32(11):e71269. doi: 10.1002/cns.71269 (PMC13761299; doi:10.1002/cns.71269)
Supplement: Supplementary file 1 [file CNS-32-e71269-s002.docx]

**2 Materials and Methods**

**2.3 EEG data acquisition**

The recordings were performed in a quiet room with moderate lighting and a comfortable ambient temperature to minimize environmental interference. Noise-reducing earplugs were used during the recording to reduce external auditory interference. EEG acquisition parameters were set as follows: sampling rate of 1000 Hz, low-pass filter at 250 Hz, and high-pass filter at 0.016 Hz (time constant of 10 seconds). The FCz electrode was used as the reference, and electrode impedance was kept below 10 kΩ throughout the recording. Resting-state EEG data consisted of 6 minutes with eyes open and 6 minutes with eyes closed. Participants were instructed to stay relaxed keep their heads still, and keep their minds blank during the recording.

**2.4 EEG data processing**

**2.4.1 EEG data preprocessing**

First, the TP9 and TP10 electrodes were set as new reference electrodes, and the original reference electrode was reassigned to FCz. A finite impulse response (FIR) band-pass filter ranging from 0.5 Hz to 45 Hz was applied to remove slow drifts and high-frequency noise, and a notch filter at 50 Hz was used to eliminate power line interference. The data were then downsampled from 1000 Hz to 500 Hz and segmented into 2-second epochs. If more than 20% of the data from a single channel was marked for rejection, that channel was replaced by spherical interpolation to preserve the integrity and accuracy of the data. To further clean the data, independent component analysis was conducted on the preprocessed data to identify and remove artifacts related to eye blinks, eye movements, muscle activity, and cardiac signals. Finally, a post-ICA quality check was performed, and any segment with EEG amplitude exceeding ±80 μV was excluded from further analysis.

**2.4.2 Source localization**

First, the preprocessed EEG data were imported into Brainstorm and co-registered with the electrode positions. The electrode coordinates were then aligned and corrected using the standard head model based on the Montreal Neurological Institute ICBM152 template. An OpenMEEG boundary element method was used to construct the head model and account for conductivity differences among the scalp, skull, and brain tissues. During forward modeling, the electric field generated by each cortical source was projected to the scalp electrodes to construct the lead field matrix, which characterizes the relationship between cortical sources and sensor measurements. Subsequently, cortical source activity was reconstructed using the Minimum Norm Imaging algorithm. A distributed cortical source model consisting of equivalent current dipoles was used, with dipoles located at cortical source vertices and their orientations constrained to be normal to the local cortical surface. The cortical source space consisted of 15,002 vertices. The inverse solution estimated distributed cortical current density by minimizing the L2-norm of source activity, and the reconstructed quantity therefore represented cortical current density at each source location.

The resulting cortical current density maps were parcellated into 68 cortical regions according to the Desikan–Killiany atlas. Network nodes were defined at the ROI level rather than at individual source vertices. Reconstructed source activity within each Desikan–Killiany parcel was summarized to obtain one regional time series for subsequent functional connectivity analysis. The full list of the 68 cortical regions is provided in the supplementary Excel file “Supplementary materials_Desikan-Killiany_68_Regions.xlsx”.

**2.4.4 Graph theory analysis**

**The global properties in the graph theoretical analysis**

**Small-worldness (Sigma):** Small-worldness refers to the property of a network in which most nodes are not directly connected to each other but can be reached through a small number of steps. It is quantified as the ratio of the normalized clustering coefficient (Gamma) to the normalized characteristic path length (Lambda). A small-world network has a high clustering coefficient and a short path length, indicating efficient communication and integration within the network. A value of Sigma > 1.1 typically indicates that the network exhibits small-world properties.

**Normalized Clustering Coefficient (Gamma):** The clustering coefficient measures the degree to which nodes in a network tend to cluster together. The normalized clustering coefficient (Gamma) compares the actual clustering coefficient of the brain network to that of a random network with the same number of nodes and edges. A higher Gamma indicates a more pronounced tendency for local clusters of nodes to form, reflecting the brain's capacity for specialized communication within regions.

**Normalized Characteristic Path Length (Lambda):** The characteristic path length measures the average number of steps required for information to travel between any two nodes in the network. The normalized characteristic path length (Lambda) compares the actual path length to that of a random network. A lower Lambda indicates that the network is more efficient in connecting distant regions, suggesting efficient communication within the brain.

**Clustering Coefficient (Cp):** The clustering coefficient measures the tendency for nodes in the network to form clusters or groups. In brain networks, higher clustering suggests that brain regions may be more specialized in certain functions and more interconnected within specific regions.

**Characteristic Path Length (Lp):** The characteristic path length is the average shortest path between all pairs of nodes in the network. In the brain, a shorter path length indicates more efficient communication between brain regions, while a longer path length suggests a less efficient network.

**Global Efficiency (Eglob):** Global efficiency measures the efficiency of information transfer across the entire network. It is calculated by considering the shortest path lengths between all pairs of nodes. Higher global efficiency suggests that the brain network can efficiently exchange information across long distances.

**Local Efficiency (Eloc):** Local efficiency measures the efficiency of information transfer within local clusters of the network. It reflects the ability of a brain region to integrate information from its neighboring regions. Higher local efficiency indicates better localized processing and integration within the brain.

**Assortativity:** Assortativity measures the tendency for nodes with similar degrees to be connected in the network. Positive assortativity indicates that highly connected regions tend to link to other highly connected regions, while negative assortativity suggests that regions with fewer connections tend to link to highly connected regions.

**Hierarchy:** Hierarchy measures the degree to which a network is organized in a hierarchical manner, where higher-level nodes control the flow of information to lower-level nodes. A more hierarchical network suggests a structured organization that allows for efficient integration of information across different levels of the brain.

**Synchronization:** Synchronization refers to the degree of temporal coordination between brain regions. In brain networks, synchronization reflects the ability of regions to function together in a coordinated manner, which is crucial for efficient cognitive processing.

**The nodal properties in the graph theoretical analysis**

**Degree Centrality (DC):** Degree centrality measures the number of direct connections a node (brain region) has with other nodes in the network. In the context of brain networks, a higher degree centrality indicates that a brain region is more connected and plays a more prominent role in communication within the network. It reflects the importance of a region in terms of its local connectivity.

**Betweenness Centrality (BC):** Betweenness centrality quantifies the number of shortest paths that pass through a node. It reflects how much control a node has over the flow of information within the network. A node with high betweenness centrality acts as a bridge or hub between different parts of the network, which can be crucial for integrating information across different brain regions.

**Nodal Efficiency (NE):** Nodal efficiency is a measure of how efficiently a node can exchange information with the rest of the network. A higher nodal efficiency suggests that a region is better at facilitating communication within the network.

**Nodal Local Efficiency (NLE):** Nodal local efficiency measures the efficiency of information transfer within a local neighborhood of a node. It is calculated by considering the local connections of a node and how efficiently they can communicate with one another. High nodal local efficiency suggests that a brain region efficiently integrates information from its direct neighbors, which is important for specialized processing.

**Nodal Shortest Path Length (Np):** Nodal shortest path length measures the shortest distance (in terms of the number of edges) between a given node and all other nodes in the network. In the brain, a smaller nodal shortest path length indicates that a brain region can quickly communicate with other regions, suggesting more efficient global communication.

**Nodal Clustering Coefficient (NCp):** Nodal clustering coefficient quantifies the extent to which a node's neighbors are also directly connected to each other. High nodal clustering indicates that a region's neighbors are well-connected, reflecting a high degree of localized integration within the network. This can be indicative of specialized or modular brain processing within a particular region.

**3 Findings**

**3.1 Baseline stage**

**3.1.1 Demographic data and clinical characteristics**

**Supplementary Table 1.** The demographic characteristics, clinical information of all participants.

|  | **HC (n = 43)** | **PD (n = 52)** | **F/Z value** | ***P value*** |
| --- | --- | --- | --- | --- |
| Gender (M/F) | 10/33 | 18/34 | 1.461^a^ | 0.227 |
| Age (years） | 49 (45, 51) | 43 (31.25, 54) | -1.475^b^ | 0.140 |
| Education level (years） | 12 (9, 15) | 12 (9, 16) | -1.849^b^ | 0.064 |
| Illness duration (years） | N/A | 4.5 (1, 36) | N/A | N/A |
| HAMA | 1 (0, 2) | 17 (13, 22.75) | -8.385^b^ | **< 0.001*** |
| PDSS | N/A | 9 (7, 12) | N/A | N/A |

Abbreviations: HAMA, Hamilton Anxiety Scale; HC, healthy control; PD, panic disorder; PDSS, Panic Disorder Severity Scale.

^a^ Chi-square test.

^b^ Mann-Whitney U test.

^*^ The results were statistically significant (*P* < 0.05).

**3.1.2 Functional connectivity**

**Supplementary Table 2.** Group Differences in Cortical Functional Connectivity between PD and HC

| **Functional connectivity** | | **T value** | ***P* value** |
| --- | --- | --- | --- |
| **α band** |  |  |  |
| Superior parietal cortex.L | Temporal pole.L | 3.626 | **< 0.001*** |
| Temporal pole.L | Caudal anterior-cingulate cortex.R | 3.448 | **< 0.001*** |
| Superior frontal gyrus.L | Rostral middle frontal gyrus.R | 3.637 | **< 0.001*** |
| Superior parietal cortex.L | Rostral middle frontal gyrus.R | 3.710 | **< 0.001*** |
| **θ band** |  |  |  |
| Isthmus-cingulate cortex.L | Rostral middle frontal gyrus.L | -3.521 | **< 0.001*** |
| Rostral anterior cingulate cortex.L | Insular cortex.L | -3.518 | **< 0.001*** |
| Superior frontal gyrus.L | Insular cortex.L | -4.078 | **< 0.001*** |
| Pericalcarine cortex.L | Isthmus-cingulate cortex.R | -3.688 | **< 0.001*** |
| Medial orbitofrontal cortex.L | Lateral orbitofrontal cortex.R | -3.423 | **< 0.001*** |
| Medial orbitofrontal cortex.L | Parahippocampal gyrus.R | -3.429 | **< 0.001*** |
| Insular cortex.L | Parahippocampal gyrus.R | -3.768 | **< 0.001*** |
| Medial orbitofrontal cortex.L | Frontal pole.R | -3.405 | **< 0.001*** |
| **δ band** |  |  |  |
| Lateral orbitofrontal cortex.L | Rostral anterior cingulate cortex.L | -3.566 | **< 0.001*** |
| Medial orbitofrontal cortex.L | Caudal middle frontal gyrus.R | -3.497 | **< 0.001*** |
| Rostral anterior cingulate cortex.L | Caudal middle frontal gyrus.R | -3.415 | **< 0.001*** |
| Inferior temporal gyrus.R | Paracentral lobule.R | -4.275 | **< 0.001*** |

^*^ NBS corrected: edge-level *P* < 0.001; subnetwork-level *P* < 0.05.

Note: Positive T-values indicate increased functional connectivity, while negative T-values indicate decreased functional connectivity.

**3.1.3 Graph theory analysis**

**Supplementary Table 3.** Group Differences in Global Topological Properties of Cortical Functional Network between PD and HC

| **Graph measures** | **Mean ± SD** | | **T value** | ***P value*** |
| --- | --- | --- | --- | --- |
|  | **HC group** | **PD group** |  |  |
| **α band** |  |  |  |  |
| Assortativity | -0.286 ± 1.290 | -0.619 ± 1.360 | -1.215 | 0.227 |
| Hierarchy | -0.669 ± 0.641 | -0.741 ± 0.650 | -0.537 | 0.592 |
| Synchronization | -0.125 ± 0.270 | -0.067 ± 0.141 | 1.344 | 0.182 |
| Global efficiency | 0.243 ± 0.019 | 0.235 ± 0.020 | -2.120 | **0.037*** |
| Local efficiency | 0.262 ± 0.016 | 0.260 ± 0.015 | -0.669 | 0.505 |
| Clustering coefficient | 0.182 ± 0.020 | 0.183 ± 0.021 | 0.276 | 0.783 |
| Normalized clustering coefficient | 0.388 ± 0.042 | 0.373 ± 0.039 | -1.821 | 0.072 |
| Normalized characteristic path length | 0.444 ± 0.003 | 0.444 ± 0.004 | 0.195 | 0.846 |
| Characteristic path length | 0.880 ± 0.105 | 0.926 ± 0.106 | 2.088 | **0.040*** |
| Small-worldness | 0.385 ± 0.042 | 0.370 ± 0.038 | -1.857 | 0.066 |
| **β band** |  |  |  |  |
| Assortativity | 0.337 ± 1.120 | 0.289 ± 0.994 | -0.217 | 0.828 |
| Hierarchy | -0.172 ± 0.661 | -0.271 ± 0.667 | -0.720 | 0.473 |
| Synchronization | -0.383 ± 0.466 | -0.339 ± 0.491 | 0.444 | 0.658 |
| Global efficiency | 0.260 ± 0.010 | 0.259 ± 0.009 | -0.333 | 0.740 |
| Local efficiency | 0.272 ± 0.015 | 0.273 ± 0.013 | 0.268 | 0.789 |
| Clustering coefficient | 0.183 ± 0.018 | 0.184 ± 0.015 | 0.478 | 0.634 |
| Normalized clustering coefficient | 0.451 ± 0.040 | 0.442 ± 0.048 | -1.003 | 0.319 |
| Normalized characteristic path length | 0.446 ± 0.005 | 0.446 ± 0.004 | -0.187 | 0.852 |
| Characteristic path length | 0.799 ± 0.057 | 0.803 ± 0.057 | 0.272 | 0.786 |
| Small-worldness | 0.445 ± 0.039 | 0.436 ± 0.046 | -1.009 | 0.316 |
| **θ band** |  |  |  |  |
| Assortativity | 0.181 ± 0.783 | 0.112 ± 0.937 | -0.384 | 0.702 |
| Hierarchy | -0.411 ± 0.653 | -0.293 ± 0.581 | 0.934 | 0.353 |
| Synchronization | -0.283 ± 0.334 | -0.345 ± 0.418 | -0.783 | 0.436 |
| Global efficiency | 0.262 ± 0.011 | 0.264 ± 0.007 | 1.350 | 0.180 |
| Local efficiency | 0.257 ± 0.012 | 0.261 ± 0.014 | 1.374 | 0.173 |
| Clustering coefficient | 0.167 ± 0.015 | 0.170 ± 0.017 | 0.962 | 0.339 |
| Normalized clustering coefficient | 0.420 ± 0.042 | 0.429 ± 0.035 | 1.125 | 0.263 |
| Normalized characteristic path length | 0.445 ± 0.002 | 0.445 ± 0.004 | 0.100 | 0.921 |
| Characteristic path length | 0.790 ± 0.068 | 0.773 ± 0.044 | -1.416 | 0.160 |
| Small-worldness | 0.416 ± 0.042 | 0.425 ± 0.036 | 1.106 | 0.271 |
| **δ band** |  |  |  |  |
| Assortativity | 0.077 ± 0.498 | 0.167 ± 0.517 | 0.860 | 0.392 |
| Hierarchy | -0.243 ± 0.420 | -0.224 ± 0.369 | 0.233 | 0.816 |
| Synchronization | -0.109 ± 0.291 | -0.132 ± 0.222 | -0.434 | 0.665 |
| Global efficiency | 0.264 ± 0.005 | 0.260 ± 0.008 | -3.403 | **0.001*** |
| Local efficiency | 0.263 ± 0.012 | 0.266 ± 0.012 | 1.343 | 0.182 |
| Clustering coefficient | 0.174 ± 0.014 | 0.181 ± 0.017 | 2.045 | **0.044*** |
| Normalized clustering coefficient | 0.426 ± 0.019 | 0.427 ± 0.019 | 0.201 | 0.841 |
| Normalized characteristic path length | 0.442 ± 0.002 | 0.442 ± 0.001 | -1.031 | 0.305 |
| Characteristic path length | 0.767 ± 0.022 | 0.787 ± 0.033 | 3.453 | **0.001*** |
| Small-worldness | 0.424 ± 0.019 | 0.425 ± 0.019 | 0.247 | 0.806 |

^*^ The results were statistically significant (*P* < 0.05).

Note: Positive T-values indicate enhanced network topological properties, while negative T-values indicate reduced network topological properties.

**3.1.5 SVM**

The top 20 features with the largest effect sizes were as following:

1) functional connectivity between left isthmus-cingulate cortex and left rostral middle frontal gyrus in the θ band,

2) functional connectivity between left medial orbitofrontal cortex and right parahippocampal gyrus in the θ band,

3) functional connectivity between left rostral anterior cingulate cortex and left insular cortex in the θ band,

4) functional connectivity between left superior parietal cortex and right rostral middle frontal gyrus in the α band,

5) functional connectivity between left pericalcarine cortex and right isthmus-cingulate cortex in the θ band,

6) functional connectivity between left insular cortex and right parahippocampal gyrus in the θ band,

7) functional connectivity between left temporal pole and left caudal anterior-cingulate cortex in the α band,

8) functional connectivity between left superior frontal gyrus and left insular cortex in the θ band,

9) functional connectivity between right inferior temporal gyrus and right paracentral lobule in the δ band,

10) functional connectivity between left medial orbitofrontal cortex and right frontal pole in the θ band,

11) functional connectivity between left superior frontal gyrus and right rostral middle frontal gyrus in the α band,

12) functional connectivity between left rostral anterior cingulate cortex and right caudal middle frontal gyrus in the δ band,

13) functional connectivity between left lateral orbitofrontal cortex and left rostral anterior cingulate cortex in the δ band,

14) functional connectivity between left medial orbitofrontal cortex and right caudal middle frontal gyrus in the δ band,

15) clustering coefficient of cortical functional network in the δ band,

16) functional connectivity between left superior parietal cortex and left temporal pole in the α band,

17) betweenness centrality of right inferior temporal gyrus in the δ band,

18) betweenness centrality of left superior frontal gyrus in the δ band,

19) nodal efficiency of left fusiform gyrus in the θ band,

and 20) betweenness centrality of left pars opercularis of inferior frontal gyrus in the δ band.

**3.2 Longitudinal follow-up stage**

**3.2.1 Clinical characteristics**

**Supplementary Table 4.** Pre- and Post-treatment Clinical Data in PD Patients

|  | **Pre-treatment Group** | **Post-treatment Group** | **Z value** | ***P* value** |
| --- | --- | --- | --- | --- |
| HAMA | 21 (14, 28) | 4 (2, 7) | -6.482 ^a^ | **< 0.001*** |
| PDSS | 12 (10, 14) | 0 (1, 2) | -6.132 ^a^ | **< 0.001*** |

Abbreviations: HAMA, Hamilton Anxiety Scale; PDSS, Panic Disorder Severity Scale.

^a^ Wilcoxon signed-rank test.

^*^ The results were statistically significant (*P* < 0.05).

**3.2.2 Functional connectivity**

**Supplementary Table 5.** Group Differences in Cortical Functional Connectivity Between Pre- and Post-Treatment in PD Patients

| **Functional connectivity** | | **T value** | ***P* value** |
| --- | --- | --- | --- |
| **δ band** |  |  |  |
| Medial orbitofrontal cortex.L | Insular cortex.L | 4.028 | **< 0.001*** |
| Lateral orbitofrontal cortex.R | Postcentral gyrus.R | 3.988 | **< 0.001*** |
| Medial orbitofrontal cortex.L | Precentral gyrus.R | 3.949 | **< 0.001*** |
| Lateral orbitofrontal cortex.R | Precentral gyrus.R | 3.930 | **< 0.001*** |
| Precentral gyrus.R | Superior temporal gyrus.R | 4.280 | **< 0.001*** |
| Superior temporal gyrus.L | Transverse temporal cortex.R | 4.502 | **< 0.001*** |
| Pars triangularis of inferior frontal gyrus.R | Transverse temporal cortex.R | 5.081 | **< 0.001*** |

^*^ NBS corrected: edge-level *P* < 0.001; subnetwork-level *P* < 0.05.

Note: Positive T-values indicate enhanced functional connectivity after treatment, while negative T-values indicate reduced functional connectivity after treatment.

**3.2.3 Graph theory analysis**

**Supplementary Table 6.** Differences in Global Topological Properties of the Cortical Functional Network Between Pre- and Post-Treatment in PD Patients

| **Graph measures** | **Mean ± SD** | | **T value** | ***P* value** |
| --- | --- | --- | --- | --- |
|  | **Pre-treatment Group** | **Post-treatment Group** |  |  |
| **α band** |  |  |  |  |
| Assortativity | -0.380 ± 1.140 | 0.063 ± 1.000 | 1.870 | 0.071 |
| Hierarchy | -0.864 ± 0.589 | -0.718 ± 0.540 | 1.321 | 0.196 |
| Synchronization | -0.109 ± 0.169 | -0.113 ± 0.245 | -0.098 | 0.922 |
| Global efficiency | 0.233 ± 0.019 | 0.244 ± 0.019 | 3.562 | **0.001*** |
| Local efficiency | 0.259 ± 0.015 | 0.264 ± 0.022 | 1.435 | 0.162 |
| Clustering coefficient | 0.184 ± 0.022 | 0.185 ± 0.028 | 0.159 | 0.875 |
| Normalized clustering coefficient | 0.371 ± 0.034 | 0.386 ± 0.039 | 2.802 | **0.009*** |
| Normalized characteristic path length | 0.444 ± 0.004 | 0.446 ± 0.004 | 1.068 | 0.294 |
| Characteristic path length | 0.935 ± 0.098 | 0.885 ± 0.110 | -2.759 | **0.010*** |
| Small-worldness | 0.367 ± 0.032 | 0.382 ± 0.039 | 2.739 | **0.010*** |
| **β band** |  |  |  |  |
| Assortativity | 0.477 ± 1.109 | -0.125 ± 0.941 | -2.428 | **0.021*** |
| Hierarchy | -0.391 ± 0.746 | -0.144 ± 0.729 | 1.233 | 0.227 |
| Synchronization | -0.254 ± 0.429 | -0.367 ± 0.396 | -1.207 | 0.237 |
| Global efficiency | 0.258 ± 0.011 | 0.258 ± 0.013 | 0.210 | 0.835 |
| Local efficiency | 0.270 ± 0.012 | 0.271 ± 0.016 | 0.561 | 0.579 |
| Clustering coefficient | 0.182 ± 0.014 | 0.182 ± 0.017 | -0.087 | 0.931 |
| Normalized clustering coefficient | 0.443 ± 0.051 | 0.439 ± 0.055 | -0.426 | 0.673 |
| Normalized characteristic path length | 0.446 ± 0.004 | 0.445 ± 0.004 | -1.020 | 0.316 |
| Characteristic path length | 0.812 ± 0.069 | 0.810 ± 0.073 | -0.130 | 0.897 |
| Small-worldness | 0.437 ± 0.050 | 0.434 ± 0.054 | -0.326 | 0.747 |
| **θ band** |  |  |  |  |
| Assortativity | 0.162 ± 1.055 | 0.128 ± 0.751 | -1.119 | 0.272 |
| Hierarchy | -0.405 ± 0.651 | -0.331 ± 0.631 | 0.146 | 0.885 |
| Synchronization | -0.342 ± 0.395 | -0.274 ± 0.333 | 0.670 | 0.508 |
| Global efficiency | 0.263 ± 0.009 | 0.261 ± 0.010 | -1.305 | 0.202 |
| Local efficiency | 0.263 ± 0.014 | 0.266 ± 0.015 | 0.873 | 0.390 |
| Clustering coefficient | 0.173 ± 0.018 | 0.177 ± 0.017 | 1.209 | 0.236 |
| Normalized clustering coefficient | 0.424 ± 0.034 | 0.425 ± 0.041 | 0.057 | 0.955 |
| Normalized characteristic path length | 0.446 ± 0.005 | 0.445 ± 0.003 | -1.250 | 0.221 |
| Characteristic path length | 0.787 ± 0.057 | 0.796 ± 0.065 | 1.063 | 0.296 |
| Small-worldness | 0.420 ± 0.034 | 0.421 ± 0.040 | 0.122 | 0.903 |
| **δ band** |  |  |  |  |
| Assortativity | 0.208 ± 0.536 | 0.112 ± 0.618 | -0.674 | 0.506 |
| Hierarchy | -0.215 ± 0.384 | -0.172 ± 0.467 | 0.369 | 0.715 |
| Synchronization | -0.069 ± 0.206 | -0.160 ± 0.385 | -1.119 | 0.272 |
| Global efficiency | 0.259 ± 0.009 | 0.261 ± 0.010 | 1.096 | 0.282 |
| Local efficiency | 0.269 ± 0.012 | 0.265 ± 0.013 | -1.710 | 0.098 |
| Clustering coefficient | 0.184 ± 0.016 | 0.178 ± 0.020 | -1.971 | 0.058 |
| Normalized clustering coefficient | 0.428 ± 0.018 | 0.431 ± 0.023 | 0.434 | 0.668 |
| Normalized characteristic path length | 0.442 ± 0.001 | 0.442 ± 0.002 | 0.000 | 1.000 |
| Characteristic path length | 0.795 ± 0.038 | 0.785 ± 0.045 | -1.110 | 0.276 |
| Small-worldness | 0.426 ± 0.018 | 0.429 ± 0.023 | 0.424 | 0.674 |

^*^ The results were statistically significant (*P* < 0.05).

Note: Positive T-values represent enhanced network topological properties after treatment, while negative T-values represent reduced network topological properties after treatment.
